# Efficacy of intensity-modulated radiotherapy with concurrent carboplatin in nasopharyngeal carcinoma

**DOI:** 10.2478/raon-2014-0044

**Published:** 2015-03-25

**Authors:** Anussara Songthong, Chakkapong Chakkabat, Danita Kannarunimit, Chawalit Lertbutsayanukul

**Affiliations:** Division of Radiation Oncology, Department of Radiology, Faculty of Medicine, Chulalongkorn University, King Chulalongkorn Memorial Hospital, Pathumwan, Bangkok, Thailand

**Keywords:** intensity-modulated radiotherapy (IMRT), carboplatin, nasopharyngeal carcinoma

## Abstract

**Background.:**

The aim of the prospective phase II study was to evaluate the efficacy and toxicities of concurrent carboplatin with intensity-modulated radiotherapy (IMRT) in the treatment of nasopharyngeal carcinoma (NPC).

**Patients and methods.:**

Between October 2005 and November 2011, 73 stage II–IVB NPC patients received IMRT 70 Gy concurrently with three cycles of carboplatin (AUC 5) every three weeks, followed by three cycles of adjuvant carboplatin (AUC 5) and 5-FU (1,000 mg/m^2^/day for four days) every four weeks. All patients were evaluated for tumour response using response evaluation criteria in solid tumour (RECIST) criteria, survival analysis using Kaplan-Meier methods, and toxicities according to common terminology criteria for adverse events (CTCAE) version 4.0.

**Results.:**

At three months after chemoradiation, 82.2% and 17.8% of patients achieved complete and partial response, respectively. With a median follow-up of 48.1 months (1.3–97.8 months), 9.6% and 17.8% had local recurrence and distant metastasis, respectively. The median survival was not reached. A three-year overall survival was 83.6% and a progression-free survival was 65.3%. Regarding treatment compliance, 97.2%, 68.5% and 69.8% completed radiation treatment, concurrent carboplatin and adjuvant chemotherapy, respectively. Grade 3–4 acute toxicities were oral mucositis (16.4%), dysphagia (16.4%), xerostomia (15.1%) and haematotoxicity (6.8%).

**Conclusions.:**

Carboplatin concurrently with IMRT provided excellent tumour response, manageable toxicities and good compliance. This should be considered as an alternative treatment for NPC patients.

## Introduction

Nasopharyngeal carcinoma (NPC) is one of the most common head and neck neoplasms among Asian people. The overall incidence of NPC in Southeast Asia is 6.5 and 2.6 per 100,000 person-years in males and females, respectively.^1^ In Thailand, the age-standardized incidence rates of NPC are approximately 3.7 and 1.2 per 100,000 in males and females, respectively.^2^

Meta-analysis showed that chemotherapy plays an important role in the treatment of this disease.^3^ Al Saraff *et al*. (Intergroup 0099) demonstrated significant benefits of additional cisplatin in terms of both disease free survival and overall survival, when used concurrently with radiation followed by a combination of cisplatin and 5-fluorouracil chemotherapy for three cycles.^4^ Thus, this regimen has become standard of care for nasopharyngeal carcinoma despite the low compliance rate (55–63%) in this trial. Significant side effects of cisplatin include nausea and vomiting, renal, neurological and ototoxicity. Additionally, during high-dose cisplatin administration, adequate hydration and volume monitoring are needed and require hospital admission. Recently, Chan *et al*. proposed a low-dose weekly cisplatin that could be administered in an outpatient setting and provides good patient compliance.^5^

Based on similar radiosensitizing properties of carboplatin and cisplatin along with pre-clinical data that demonstrated an enhanced radiation effect from concurrent carboplatin in tumour cells, some physicians use carboplatin as an alternative regimen to avoid serious cisplatin toxicities, especially renal, gastrointestinal and neurotoxicity.^6^–^10^

Many studies have shown comparable response rates and survival outcomes with acceptable toxicities and better compliance from carboplatin.^11^–^14^ However, the radiation technique in those studies was the conventional technique. More recently, intensity-modulated radiotherapy (IMRT) has been proven in NPC treatment for its efficacy and its advantages over conventional techniques and has been considered as a standard radiation technique for NPC.^15^–^17^

The objectives of this study are to evaluate efficacy and toxicities using IMRT concurrently with carboplatin, followed by adjuvant carboplatin and 5-fluorouracil (5-FU) chemotherapy for the treatment of NPC.

## Patients and methods

### Patients and methods

Between October 2005 and November 2011, newly diagnosed NPC patients were accrued for this prospective phase II study after obtaining the institutional review board approval (RA 13/49). The eligibility criteria included those aged 18 years old and above; histologically confirmed non-metastatic nasopharyngeal carcinoma stage II–IVB according to the 7^th^ edition of the American Joint Committee on Cancer Staging System (AJCC 2010); Eastern Cooperative Oncology Group (ECOG) performance status 0–2; adequate hematologic and renal function, defined by with blood cells (WBC) ≥ 4,000/mL, platelet count ≥ 100,000/mL, serum creatinine ≤ 1.5 mg/dL or calculated creatinine clearance ≥ 60 ml/min. Patients with distant metastasis; previous radiation and/or chemotherapy treatment less than six months prior to the study entry; other malignancy except non-melanoma skin cancer or a carcinoma of non-head and neck origin, controlled for at least five years; active infection; major medical or psychiatric condition or pregnancy were excluded.

All eligible patients received a pre-treatment evaluation including complete history and physical examination, endoscopic biopsy, routine laboratory tests for hematologic, renal and hepatic function as well as a dental and nutritional evaluation before the treatment. Radiological investigations consisted of computed tomography (CT) scan or magnetic resonance imaging (MRI) of the nasopharynx, chest radiography, ultrasound of upper abdomen and bone scintigraphy. Positron emission tomography (PET) scan was optional. A pathologic confirmation of NPC was performed and re-classified according to WHO subtype.^18^

### Treatment protocol

Each patient underwent contrast-enhanced CT simulation with a long thermoplastic mask. The GTVs and CTVs were contoured according to RTOG guidelines. There were two planning target volumes (PTVs): PTV-high risk (PTV-HR), defined as primary tumour and gross lymphadenopathy with appropriate margin, and PTV-low risk (PTV-LR), defined as PTV-HR plus elective lymph node region. The prescription dose was 50 Gy in 25 fractions to PTV-LR followed by a boost of 20 Gy in 10 fractions, called sequential IMRT (SEQ). Recently, a simultaneous integrated boost (SIB) technique was developed and applied in last few patients with total dose of 70 Gy and 56 Gy in 33 fractions for PTV-HR and PTV-LR, respectively. Normal tissue constraints were used according to our institutional protocol (adopted from RTOG 0225 and 0615 study protocols) and are described in Table 1.

All patients received IMRT concurrently with three cycles of carboplatin (AUC 5) every three weeks, followed by three cycles of adjuvant carboplatin (AUC 5) and 5-FU (1,000 mg/m^2^ /day for four days) every four weeks.

During the concurrent and the adjuvant treatment, patients were assessed weekly. Dose modification and proper management were performed according to patients’ toxicity grading. The response of the primary tumour and lymph node was evaluated at three months after the last fraction of radiotherapy by endoscopic examination and CT scan. Other imaging was performed if indicated.

### Statistical analysis

Data collection consisted of patient characteristics including age, sex and ECOG performance status; disease characteristics including pathologic WHO subtype and TNM staging; and treatment modalities including radiation treatment technique, radiation dose, duration of radiation treatment as well as compliance with radiation and chemotherapy. All patients were evaluated for tumour response using response evaluation criteria in solid tumour (RECIST) criteria, survival outcomes using Kaplan-Meier methods, and acute and late toxici-ties according to common terminology criteria for adverse events (CTCAE) version 4.0.

Primary endpoints were progression-free survival (PFS) and overall survival (OS). PFS was defined as the time period since the initial treatment of NPC until disease recurrence or progression or death. OS was defined as the time period between the initial treatment of NPC and any cause of death. Survival analyses were computed using the Kaplan-Meier method and log-rank test. P-value of 0.05 or less was applied to define significance. Statistical Packages for Social Sciences (SPSS) software version 17.0 was used for the statistical analysis. Secondary endpoints were disease control and treatment-related toxicities.

The sample size calculation was based on the proportion of expected death (mortality rate) with 95% confidence interval. We employed 18.1% mortality rate for concurrent radiation with carboplatin according to the results of a randomized study of Chitapanarux *et al*.^13^ Allowing 20% dropout p-value of 0.05, 69 participants were planned to be enrolled in the study.

## Results

### Patient and disease characteristics

A total of 73 patients diagnosed with NPC and treated between October 2005 and November 2011 were accrued. Patient and disease characteristics are listed in Table 2. The mean age was 54.4 years (range 24–76 years). The majority of patients (67.1%) were males. All were in good performance status and had non-serious comorbidities. The histological subtype, according to WHO classification, was non-keratinizing squamous cell carcinoma (NK-SCCA) in every patient, which could be further identified as undifferentiated NK-SCCA in most patients (83.6%). Approximately half of patients had stage III disease.

### Radiation treatment

Whole-neck IMRT was planned using the Eclipse treatment planning system. The majority of patients (91.8%) was treated with the SEQ-IMRT technique. The rest were treated with the SIBIMRT technique. Seventy-two patients (98.6%) completed a course of radiation. One patient could not complete the course of radiation and the treatment interruption of 38 days occurred in another patient; both resulted from intolerable toxicity. The average PTV-HR and PTV-LR dose were 69.95 Gy (range 58–76 Gy) and 50.82 Gy (range 42–62 Gy), respectively. The median duration of the radiation treatment was 55 days (range 14–93 days).

### Chemotherapy

Seventy-two patients (98.6%) received concurrent carboplatin and radiation; 50 patients (68.5%) received all three cycles of chemotherapy as planned. The compliance of chemotherapy treatment is detailed in Table 3.

### Clinical outcome

The median follow-up time was 48.1 months (6.1–97.8 months). At three months after completion of radiotherapy, a complete response (CR) was achieved in 60 patients (82.2%) while 13 patients (17.8%) achieved a partial response (PR). Regarding the site of the tumour response, 94.5% of patients achieved CR at the primary (nasopharyngeal) site while 83.6% achieved CR at regional lymph node sites. Patients who achieved PR received a further treatment: a radiotherapy boost of 30 Gy in 10 fractions to any residual disease at the primary site. Patients with small residual lymph node(s) had a radiation boost of 15 Gy in 5 fractions while those with a larger residual disease in the neck underwent a salvage neck dissection. No additional systemic therapy was given after the patients completed three chemotherapy cycles.

During the follow-up period, seven patients (9.6%) and 13 patients (17.8%) experienced local and distant failure, respectively. None of the patients had regional recurrence or both, local/regional and distant failure. The median time to local and distant recurrence was 20.3 months and 22.2 months, respectively. The most common sites of metastasis were bone (8.2%), liver (6.8%) and lung (4.1%).

At the last follow-up, 45 patients (61.6%) were alive without disease while eight patients (10.9%) had disease recurrence. There were 20 deaths (27.4%); 14 patients died from the progression of the disease.

### Survival outcome

**FIGURE 1.**
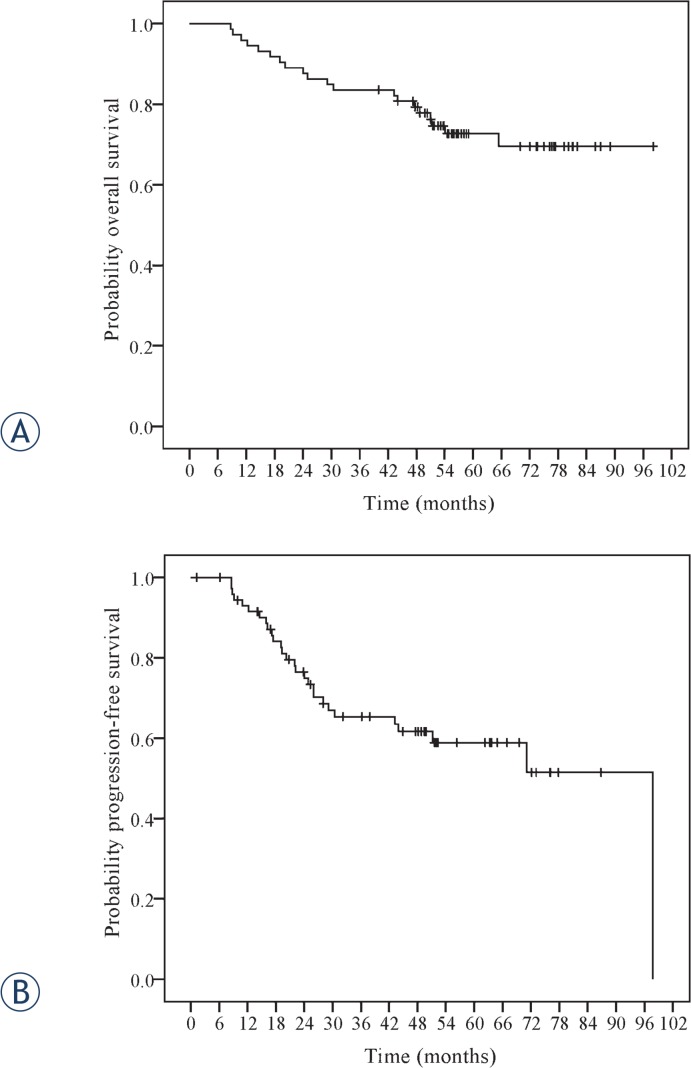
Overall and progression-free survival of patients with nasopharyngeal carcinoma treated with intensity-modulated radiotherapy and concurrent carboplatin.

Median OS was not reached. Median PFS was 71 months. Three-year OS and PFS were 83.6% and 65.3%, respectively and at 5 years they were 72.7% and 58.9%, respectively, as shown in Figure 1.

### Toxicities

The toxicities were classified as acute and late toxicities using a 90-day cut-off point after the completion of chemoradiation. Acute toxicity consisted of symptoms developing during the concurrent and the adjuvant treatment. During concurrent chemo-radiation, all patients experienced some degrees of acute toxicities, most of which were mild (grade 1–2). The most common grade 3–4 toxicities were mucositis (16.4%), dysphagia (16.4%) and xerostomia (15.1%). Only two patients (2.7%) had severe nausea and vomiting. Twelve patients (16.4%) needed nasogastric tube insertion. Weight loss of more than 20% (grade 3) occurred in 5 patients (6.8%) during concurrent chemoradiation and in 24 patients (32.9%) during the adjuvant period. During adjuvant chemotherapy, most patients recovered from mucositis, xerostomia and dysphagia. Grade 3 or more hematologic toxicities developed in 6 patients (8.2%). Three (4.1%) and two patients (2.7%) developed grade 3–4 neutropenia and thrombocytopenia, respectively. A renal function impairment was not found. No patient developed grade 5 toxicity during the concurrent and the adjuvant treatment. The incidence of acute toxicities is described in Table 4.

Eighteen patients (28.6%) had grade 3 weight loss at one year after chemoradiation. Most patients regained their weight within two years. Grade 2 xerostomia was found in 10 patients (13.7%) and three patients (4.1%) at 6-month and 12-month follow-up, while no patient had grade 2 xerostomia at the 24-month point. None of the patients experienced grade 3–4 gastrointestinal and dermatologic toxicities during follow-up. There was no renal toxicity among these patients.

## Discussion

Nasopharyngeal carcinoma is one of the most common head and neck cancer in Southeast Asia and has a different natural history and prognosis from other cancers in this region. The current standard treatment of locally advanced NPC is concurrent chemoradiation followed by adjuvant chemotherapy.^3^–^5^ According to a meta-analysis from eight trials involving 1,753 patients, chemotherapy resulted in an absolute survival benefit of 6% (from 56% to 62%) and an event-free survival benefit of 10% (from 42% to 52%) at five years. This study also concluded that the concurrent trials showed significant survival benefit with hazard ratio (HR) of 0.60 (95% CI, 0.48–0.76).^3^ Another meta-analysis from 10 randomized clinical studies with a total of 2,450 patients supported that concurrent chemo-radiation improved survival by 20% at five years with a pooled HR of 0.48 (95% CI, 0.32–0.72).^19^ The landmark study by Al Saraff *et al.* (INT 0099)4 supported the standard treatment of nasopharyngeal carcinoma using concurrent radiation with cisplatin followed by cisplatin and 5FU with 5-year OS and DFS of 67% and 58%, respectively. Although cisplatin is a widely accepted regimen, its toxicity, including nausea, vomiting, ototoxicity, neurotoxicity and nephrotoxicity, may lead to poor compliance; only 63% and 55% of patients completed concurrent and adjuvant chemotherapy in INT 0099.^4^

Carboplatin has come into interest due to its lesser side effects, especially gastrointestinal and nephrogenic side effects. The advantages of carboplatin are tolerable toxicity leading to better compliance and its capability of out-patient administration, thus reducing hospitalization, cost of the treatment and workload of medical personnel. Eisenberger indicated that 100 mg/m^2^/week of carboplatin was well tolerated when given concurrently with radiation in locally advanced head and neck cancers.^20^ Unfortunately for NPC, a prospective phase I/II study from Canada of concurrent carboplatin in 47 patients reported probably inferior OS and PFS with acceptable toxicity compared to INT 0099. With the median follow-up of 23.1 months, 3-year OS and PFS were 56% and 58%, respectively.^11^ Nevertheless, different WHO histological subtypes may result in different natural history of disease and response between Caucasian and Asian people. The randomized controlled trial from Thailand, comparing carboplatin-based chemotherapy with an INT 0099 regimen in 220 patients demonstrated a non-inferior survival outcome. Three-year OS and DFS in the carboplatin arm were 79.2% and 60.9% compared with 77.7% and 63.4% in the cisplatin arm. They also showed better compliance to treatment in carboplatin arm, 73% versus 59%.^13^ Another report from Thailand that included 50 patients using concurrent chemo-radiation with carboplatin followed by carboplatin and 5FU in NPC showed good results and tolerability. The 3-year OS and PFS were 72.7% and 89.7%, respectively.^14^

Although the efficacy of concurrent carboplatin with radiation in NPC was demonstrated, these studies used a conventional radiation technique^11^,^13^,^14^ whereas in current practice, the standard radiation technique for NPC is IMRT, which has demonstrated significantly better salivary flow rate and quality of life in NPC patients compared with conventional techniques.^15^–^17^ Additionally, the recent RTOG phase II trial 0225 and a prospective study from Memorial Sloan Kettering Cancer Center (MSKCC) reported promising results of tumour control and toxicities by using IMRT and concurrent cisplatin.^21^,^22^ In RTOG 0225 with 68 patients, 2-year OS and PFS were 80.2% and 72.7%, respectively. In the MSKCC study with 74 patients, 3-year OS and PFS were 83% and 67%, respectively. In this study, using concurrent carboplatin with IMRT, our 3-year OS and PFS were 83.6% and 65.3%, which were comparable with those previous studies. Moreover, approximately 70% of patients completed three cycles of adjuvant chemotherapy.

Distant metastasis is the major reason of failure in nasopharyngeal carcinoma. The rate of distant metastasis reported in many series was 14.7%–22% in cisplatin-based chemotherapy series compared with 14.3%–29.8% in carboplatin-based series.^4^,^11^–^14^,^18^,^21^,^22^ In our study, the crude distant metastasis rate was 17.8%, which was comparable with the results from studies using either of the two chemotherapy regimens. The most common sites of metastasis were bone (8.2%), liver (6.8%) and lung (4.1%), giving the 3- and 5-year distant metastasis-free survival (DMFS) of 82% and 77.1%, respectively.

With regards to toxicity, because different criteria were used for evaluation, comparing the toxicity among several published trials including our study might not be appropriate. Compared with RTOG 0225, which used similar toxicity evaluation criteria, Common Terminology Criteria for Adverse Events (CTCAE), overall acute grade 3 toxicity was 61.8%^21^, while it was 24.7% in our study. Acute grade 3 mucositis, defined as confluent pseudomembranous reaction (contiguous patches generally > 1.5 cm in diameter) in CTCAE version 2.0 and as severe pain or interference with oral intake in CTCAE version 4.03, was 29.4% in the RTOG study compared to 16.4% in our study. There was no patient who experienced acute grade 5 mucositis in this trial but one patient (1.3%) did in the RTOG study. According to Dechapunkul, using conventional radiation and RTOG acute toxicity criteria in which grade 3 mucositis was defined as confluent fibrinous mucositis or severe pain requiring narcotics, grade 3–4 mucositis was reported in 42% of total 50 patients.^14^ In contrast, in the Chitapanarux study, using conventional radiation technique and RTOG acute toxicity criteria, the rate of mucous membrane toxicity was very low: 0% in the cisplatin arm and 5% in the carboplatin arm. However, the rate of nasogastric tube insertion was as high as 48% and 22% in the cisplatin and carboplatin arm, respectively, compared with 16.4% in our study.

Grade 3 or higher nausea and vomiting rates during chemoradiation in our study were comparable to those of carboplatin studies and was less than cisplatin studies, for example, 2.7% in our study, 0% in Chitapanarux study, 8% in Dechapunkul study versus 19.2% in INT 0099 study.

The rate of grade 3 dermatitis, which was defined as confluent moist desquamation ≥ 1.5 cm diameter and not confined to skin folds or pitting oedema in CTCAE version 2.0 and RTOG toxicity criteria and as moist desquamation other than skin folds and creases or bleeding induced by minor trauma or abrasion in CTCAE 4.03, was 13.2% in the RTOG 0225 study, 3% and 6% in the cisplatin and carboplatin arm in the Chitapanarux study, and none in our study.

Late grade 2 xerostomia at 12-month period in our study was 4.1% compared with 13.5% reported in RTOG 0225 and 24% in the Chitapanarux study.

The treatment schedule, compliance and outcome in each study are demonstrated in Table 5.

One of the major concerns of carboplatin administration is hematologic toxicity. In our study, 8.2% experienced grade 3–4 hematologic toxicity, mainly neutropenia (6.8%) and thrombocytopenia (1.4%), which was comparable to the Chitapanarux study in which 10% and 2% of patients experienced grade 3 neutropenia and thrombocytopenia in the carboplatin arm.

This is the first study to our knowledge that demonstrated the efficacy and feasibility of carboplatin concurrently with IMRT in NPC and that this treatment can be applied as an alternative chemotherapy regimen, especially in vulnerable patients or those not suitable for standard cisplatin regimen.

## Conclusions

Carboplatin concurrently with IMRT provided excellent tumour response, manageable toxicities and good compliance. This should be considered as alternative to standard treatment with cisplatin for NPC patients.

## Figures and Tables

**TABLE 1.** Dose volume constraints of normal tissue

**Organ at risk**	**Maximum dose (Gy)**	**Dose volume constraints**	
		**Dose (Gy)**	**Maximum volume**
Spinal cord	50	45	1 cc
Brain stem	60	54	1 cc
One parotid gland		26	50%
Optic nerve	54		
Cochlear		46	50%
Eyes	24		50%
Lens	6		
Mandible	70	53	50%
Oral cavity	60	40	50%
Vocal cord	58	45	50%

Maximum dose (Dmax) defined as radiation dose encompasses 1% of each organ-at-risk volume

**TABLE 2.** Patients and disease characteristics

	**N (73)**	**%**
Age, years		
Mean (range)	54.4 (24–76)	
Sex		
Male	49	67.1%
Female	24	32.9%
Performance status		
ECOG 0	67	91.8%
ECOG 1	6	8.2%
WHO classification		
Type II (Non-keratinizing SCCA)	73	100.0%
T stage		
1	15	20.6%
2	26	35.6%
3	23	31.5%
4	9	12.3%
N stage		
0	5	6.8%
1	19	26.0%
2	41	56.2%
3a	5	6.9%
3b	3	4.1%
M stage		
0	73	100.0%
Stage grouping		
II	16	22.0%
III	42	57.5%
IV A	12	16.4%
IV B	3	4.1%

SCCA = Squamous cell carcinoma

**TABLE 3.** Compliance of chemotherapy treatment

**Number of cycles**	**N (%)**	
	**Concurrent carboplatin**	**Adjuvant carboplatin/5-FU**
0	1 (1.4%)	4 (5.5%)
1	2 (2.7%)	8 (11%)
2	20 (27.4%)	10 (13.7%)
3	50 (68.5%)	51 (69.8%)
Total	73 (100%)	73 (100%)

**TABLE 4.** Acute toxicity during treatment

**Toxicities**	**Acute toxicity During chemoradiation**		**Acute toxicity During adjuvant period**	

	**0–2**	**3–5**	**0–2**	**3–5**
Constitutional symptoms				
Fatigue	72 (98.6%)	1(1.4%)	71(97.3%)	2(2.7%)
Anorexia	71(97.3%)	2(2.7%)	73(100%)	0
Weight loss	68(93.2%)	5(6.8%)	49(67.1%)	24(32.9%)
Gastrointestinal				
Oral mucositis	61(83.6%)	12(16.4%)	73(100%)	0
Xerostomia	62(84.9%)	11(15.1%)	73(100%)	0
Dysphagia	61(83.6%)	12(16.4%)	73(100%)	0
Nausea	71(97.3%)	2(2.7%)	73(100%)	0
Vomiting	71(97.3%)	2(2.7%)	73(100%)	0
Diar rhea	73(100%)	0	73(100%)	0
Dermatitis	73(100%)	0	73(100%)	0
Hematologic				
Anaemia	73(100%)	0	72(98.6%)	1(1.4%)
Neutropenia	68(93.2%)	5(6.8%)	70(95.9%)	3(4.1%)
Thrombocytopenia	72(98.6%)	1(1.4%)	71(97.3%)	2(2.7%)
Creatinine	73(100%)	0	73(100%)	0
Total	73(100%)	0	73(100%)	0

**TABLE 5.** Comparison of treatment schedule, compliance and outcome between studies on concurrent chemoradiation with carboplatin in NPC patients and INT 0099 trial; and RTOG 0225 using IMRT technique

**Study**		**N**	**Stage**	**F/U**	**RT**	**CMT**		**Compliance CMT**		**DFS / PFS**	**OS**

						**Concurrent**	**Adjuvant**	**Concurrent**	**Adjuvant**		
INT 0099^4^	RCT	147	III 9% IV 91%	NA	Conventional 66–70 Gy	Cis 100 mg/m^2^ q 3 wk × 3 cycles	Cis 80 mg/m^2^ +5FU 4000 mg/m^2^ q 4 wk × 3 cycles	63%	55%	58%(5Y)	67%(5Y)
Parliament^11^	Prospective phase I/II (AJCC 2002)	47	I/II 10.7% III 14.9% IV 74.5%	23.1 mo	Conventional 70 Gy	Carbo 100mg/m^2^ q 1 wk × 6 cycles	-	95.7%	-	58%(3Y)	56%(3Y)
Chitapanarux^13^	RCT (AJCC 1997)	206	III 36% IVA 25% IVB 40% III 31% IVA 23% IVB 46%	26.3 mo	Conventional 70 Gy	Cis 100 mg/m^2^ q 3 wk × 3 cycles Carbo 100mg/m^2^ q 1 wk × 6 cycles	Cis 80 mg/m^2^ +5FU 4000 mg/m^2^ q 4 wk × 3 cycles Carbo AUC 5 +5FU 4000 mg/m^2^ q 4 wk × 3 cycles	59% 77%	42% 72%	63.4%(3Y) 60.9%(3Y)	77.7%(3Y) 79.2%(3Y)
Dechaphunkul^14^	Prospective (AJCC 2002)	50	IIB 8% III 36% IVA 38% IVB 18%	37.3 mo	Conventional 66–70 Gy	Carbo AUC 6 q 3 wk × 3 cycles	Carbo AUC 5 +5FU 4000 mg/m^2^ q 3 wk × 2 cycles	98% (total 5 cycles)		89.7%(3Y)	72.7%(3Y)
RTOG 0225^21^	Prospective Phase II (AJCC 1997)	68	I 13.2% IIA 2.9% IIB 25.0% III 30.9% IVA 16.2% IVB 11.8%	31.2 mo	IMRT 70 Gy	Cis 100 mg/m^2^ q 3 wk × 3 cycles	Cis 80 mg/m^2^ +5FU 4000 mg/m^2^ q 4 wk × 3 cycles	87%	45.6%	72.7% (2Y)	80.2% (2Y)
MSKCC^22^	Prospective Phase II (AJCC 1997)	74	I 7% IIB 16% III 30% IVA/B 47%	35 mo	IMRT 70 Gy (AF and SIB)	Cis 100 mg/m^2^ q 3 wk × 2 cycles	Cis 80 mg/m^2^ +5FU 4000 mg/m^2^ q 4 wk × 3 cycles	92%	NA	67% (3Y)	83% (3Y)
This study	Prospective Phase II (AJCC 2010)	73	II 22.0% III 57.5% IVA 16.4% IVB 4.1%	48.1 mo	IMRT 70 Gy (Seq and SIB)	Carbo AUC 5 q 3 wk × 3 cycles	Carbo AUC 5 +5FU 4000 mg/m^2^ q 4 wk × 3 cycles	68.5%	69.8%	Median=71m 65.3% (3Y) 58.9% (5Y)	MS=not reached 83.6% (3Y) 72.7% (5Y)

AF = Accelerated fractionation (here, hyperfractionated concomitant boost); AJCC = American Joint Committee on Cancer Staging; Carbo = Carboplatin; Cis = Cisplatin; CMT = Chemotherapy; DFS = Disease-free survival; F/U = Follow-up time; 5FU = 5-fluorouracil; mo = months; NA = Not available; OS = Overall survival; PFS = Progression-free survival; RCT = randomized-controlled trial; RT = Radiation treatment; SIB = simultaneous integrated boost; wk = week
